# Internet of Vehicles and Cost-Effective Traffic Signal Control

**DOI:** 10.3390/s19061275

**Published:** 2019-03-13

**Authors:** Sanghyun Ahn, Jonghwa Choi

**Affiliations:** Department of Computer Science and Engineering, University of Seoul, Seoul 02504, Korea; David13@uos.ac.kr

**Keywords:** Internet of Vehicles, Internet of Things, traffic signal control, vehicle queue, vehicular communication

## Abstract

The Internet of Vehicles (IoV) is attracting many researchers with the emergence of autonomous or smart vehicles. Vehicles on the road are becoming smart objects equipped with lots of sensors and powerful computing and communication capabilities. In the IoV environment, the efficiency of road transportation can be enhanced with the help of cost-effective traffic signal control. Traffic signal controllers control traffic lights based on the number of vehicles waiting for the green light (in short, vehicle queue length). So far, the utilization of video cameras or sensors has been extensively studied as the intelligent means of the vehicle queue length estimation. However, it has the deficiencies like high computing overhead, high installation and maintenance cost, high susceptibility to the surrounding environment, etc. Therefore, in this paper, we propose the vehicular communication-based approach for intelligent traffic signal control in a cost-effective way with low computing overhead and high resilience to environmental obstacles. In the vehicular communication-based approach, traffic signals are efficiently controlled at no extra cost by using the pre-equipped vehicular communication capabilities of IoV. Vehicular communications allow vehicles to send messages to traffic signal controllers (i.e., vehicle-to-infrastructure (V2I) communications) so that they can estimate vehicle queue length based on the collected messages. In our previous work, we have proposed a mechanism that can accomplish the efficiency of vehicular communications without losing the accuracy of traffic signal control. This mechanism gives transmission preference to the vehicles farther away from the traffic signal controller, so that the other vehicles closer to the stop line give up transmissions. In this paper, we propose a new mechanism enhancing the previous mechanism by selecting the vehicles performing V2I communications based on the concept of road sectorization. In the mechanism, only the vehicles within specific areas, called sectors, perform V2I communications to reduce the message transmission overhead. For the performance comparison of our mechanisms, we carry out simulations by using the Veins vehicular network simulation framework and measure the message transmission overhead and the accuracy of the estimated vehicle queue length. Simulation results verify that our vehicular communication-based approach significantly reduces the message transmission overhead without losing the accuracy of the vehicle queue length estimation.

## 1. Introduction

Nowadays, the automobile industry is focused on developing smart vehicles equipped with various sensors, computing power and communication functionalities. The Internet of Vehicles (IoV) [1] is part of the Internet of Things (IoT) because in a broad sense smart vehicles are smart things and, in another way, smart vehicles are realized with things like various sensors. Smart vehicles can be aware of and act properly according to their surrounding situations as recognized by sensors. Vehicular communications (or vehicle-to-everything communications, V2X) are one of the necessary means for situation awareness and cooperative operations among vehicles and can be categorized into vehicle-to-vehicle (V2V), vehicle-to-infrastructure (V2I) and vehicle-to-sensor (V2S) communications [2].

The efficiency of road transportation depends heavily on the performance of traffic signal controllers. Traffic signal control systems have been rapidly evolved during the last several decades [3]. Thanks to that, the traffic handling capacity of roads is significantly improved, and travel time and fuel consumption are reduced. In these days, traffic lights can be controlled in real-time by coordinated controllers at intersections which monitor traffic patterns with the assistance of devices like video cameras and sensors (e.g., loop detectors). Video camera-based monitoring [4,5,6,7,8,9,10] requires high computing power for real-time image processing and sensor-based monitoring [11,12,13,14,15,16,17] incurs high sensor installation and maintenance cost. Moreover, these technologies suffer from various environmental obstacles like weather, lighting and road condition, which cannot be completely overcome by any countermeasures.

Therefore, in this paper, we propose to use vehicular communications for traffic signal control because the vehicular communication functionality, one of the essential capabilities of IoV, has many advantages like no additional operational (installation and maintenance) cost, lightweight computing, resilience to lighting condition (i.e., can operate all day) and resilience to harsh road condition (e.g., can operate to a certain degree even in a non-line-of-sight environment). In controlling traffic signals, we can substitute vehicular communications for video cameras and sensors, which can be achieved by making vehicles notify traffic signal controllers of their existence via V2I communications so that traffic signal controllers can estimate how many vehicles are waiting for the green light (i.e., the vehicle queue length [18,19]).

For vehicular communications, the IEEE Wireless Access in Vehicular Environments (WAVE) [20] and the IEEE 802.11p [21] are standardized. From the perspective of vehicular communications, traffic signal controllers are road side units (RSUs). The channel access performance of the IEEE 802.11p significantly deteriorates as the access attempts to the channel increases because the MAC protocol of the IEEE 802.11p is based on the carrier sense multiple access (CSMA) mechanism. In the vehicle queue of a multiple lane road, vehicles tend to line up compactly and communicate various types of data traffic via V2X communications, so the message collision possibility of the vehicles in the vehicle queue (near the intersection area) is much higher than the other areas of the road. Therefore, it is desirable to alleviate the collision possibility of messages by reducing the messages generated by vehicles for traffic signal control (i.e., the V2I messages sent to the traffic signal controller). Particularly, with considering only the V2I communications for the vehicle queue length estimation, if each vehicle in the vehicle queue attempts to send a message to the traffic signal controller (we call this the Naïve mechanism), multiple message transmissions may co-exist in the air, causing collisions, because there can be more than one vehicle joined the vehicle queue almost at the same time due to multiple lanes of a road and the stopping speed. Therefore, we need a mechanism to limit the vehicles sending messages to the traffic signal controller in order to reduce the possibility of collisions and the message transmission overhead.

In our previous work [22], we proposed a mechanism, called the distance-based mechanism, that considers the distance of a vehicle from an intersection as the criterion of controlling the message transmission to the traffic signal controller. To the best of our knowledge, [22] has addressed this issue for the first time. In the distance-based mechanism, a timer is used to determine the time for a vehicle to send a message to the traffic signal controller according to the distance from the upcoming intersection. If a vehicle overhears a transmission from another vehicle behind itself, it gives up its transmission. Thus, the distance-based mechanism reduces the message transmission overhead to a half of the Naïve mechanism. However, on the red light, vehicles tend to line up one after another with slowing down their speeds with some time gap, so they may try to send messages to the traffic signal controller sequentially even if higher preferences are given to vehicles farther away from the upcoming intersection.

In this paper, to overcome this sequential transmission characteristic of the distance-based mechanism, we propose a new mechanism that can reduce the number of messages transmitted to the traffic signal controller. This newly proposed mechanism is called the sector-based mechanism. The sector-based mechanism further reduces the number of the vehicles sending messages to the traffic signal controller by adopting the concept of sectors. There can be a number of sectors in a road segment between two consecutive intersections. A sector of a road segment is a subarea of the road segment. Instead of all the vehicles waiting for the green light having rights to perform V2I communications, only the vehicles within the sectors are allowed to transmit messages to the approaching traffic signal controller. That is, the set of candidate vehicles for sending messages to the traffic signal controller of the sector-based mechanism is smaller than that of the distance-based mechanism, resulting in less transmissions to the traffic signal controller. For the performance evaluation, intensive simulations are carried out by utilizing the vehicle network simulation framework Veins [23] based on SUMO [24] and OMNet++ [25] with considering various performance- affecting factors like sector length, inter-sector distance, vehicle density of the road segment, etc. In the performance evaluation section, we can observe that the sector-based mechanism, with the sector length 10 m and the inter-sector distance 10 m, performs almost the same as the distance-based mechanism in terms of the estimation accuracy of the vehicle queue length with significantly less V2I message transmissions, almost a third of the distance-based mechanism (i.e., a sixth of the Naïve mechanism). Because the parameters like sector length and inter-sector distance are easily adjustable, the sector-based mechanism can be a good candidate for estimating the vehicle queue length for intelligent traffic signal control in the IoV environment.

The rest of the paper is organized as follows: in Section 2, we will describe the related work on traffic pattern monitoring mechanisms. Section 3 describes the detailed operation of our V2I communication-based traffic pattern monitoring and vehicle queue estimation mechanisms. In Section 4, we evaluate the performance of our mechanisms from the intensive simulation results. Finally, Section 5 concludes this paper.

## 2. Related Work

In this section, we first go over the definition of the vehicle queue. The vehicle queue is defined as a line of the vehicles stopping at the red light and the vehicles approaching to the stopping vehicles at speeds slower than the given stopping speed in the Highway Capacity Manual [19]. In [20], the vehicle queue is composed of the standing queue and the moving queue. The standing queue is with the vehicles stopping at the red signal and the moving queue with the vehicles slower than the stopping speed because of the standing queue. The equivalent standing queue is defined as the vehicle queue including both the standing queue and the moving queue. In this paper, we adopt the equivalent standing queue of [20] as the vehicle queue.

For the estimation of the vehicle queue length, first of all, the vehicles waiting for the green signal have to be recognized, which can be accomplished by utilizing devices like video cameras mounted on fixed roadside structures such as traffic signal controllers or like sensors installed under the pavement. The time-stringent control of traffic signals requires real-time processing of video frames and the accurate measurement of vehicle queue length requires sophisticated deployment of sensors.

In the video-based approach, the first thing to do for the vehicle queue length estimation is detecting vehicles from video frames in real time. After the vehicle detection process, vehicles are tracked and counted in real time. Thanks to various computer vision techniques and hardware capabilities, real-time processing of vehicle detection, tracking and counting becomes possible [4,5,6,7,8]. The mechanisms that can be used for real-time vehicle detection from video images are background subtraction method, blob analysis, thresholding, hole filling, morphological operations, etc. Once vehicles are detected, vehicle tracking and counting are performed with using various schemes like similarity measurement, patch analysis, virtual detection line, virtual detection zone, shadow detection, removal, etc. A sequence of complex processing of video images induces very high computing overhead and requires specialized hardware to expedite the processing. In [7], ARM/FPGA processor-based vehicle counting system is proposed to expedite video processing. As an example, the video processing procedure of vehicle detection and counting proposed in [4] consists of preprocessing, background update, background subtraction, image segmentation, lamplight or shadow suppression, contour extraction and filling, vehicle detection and vehicle counting using virtual coil or detecting line depending on traffic congestion situation. Even with various video processing techniques, the adversary road surrounding environment, like bad weather (e.g., rain drops and snowflakes), dim lights, curved roads, etc., may significantly downgrade the quality of video images. The authors of [4] aimed to provide robustness to video processing for vehicle detection, tracking and counting in various weather and light conditions. [9] and [10] improve robustness and accuracy even under bad road situations by adopting a feature-based detection method and a machine learning-based method, respectively, but consume abundant resources and may not guarantee real-time processing of video frames due to processing complexity. Recently, the mechanisms based on video images from unmanned aerial vehicles (UAVs) for traffic monitoring have been studied and this UAV-based approach is appropriate for large area monitoring with overcoming obstacles from wider top-view video images. For instance, in [8], a framework based on UAVs is proposed for moving-vehicle detection, multi-vehicle tracking and vehicle counting. As we have described, most of the work on the video-based approach tackles previously-mentioned environmental hurdles which may not be completely overcome by the means of various video processing methods.

In the sensor-based approach, various types of sensors, like inductive loop detectors, ultrasonic sensors, magnetometers, radar/lidar based sensors, etc., are installed near to intersections for vehicle detection, tracking and counting [11,12,13,14,15,16,17]. Each sensor is equipped with devices like a microphone to collect acoustic, seismic or any signals to classify vehicles. From the collected sensing signals, sensors and base stations detect, track and count vehicles. However, in the harsh road environment, sensing signals are affected by ambient noise, resulting in resource-intensive signal processing. Typical road sensors are deployed under the road surface at specific points and monitor the presence of vehicles at fixed locations, separately in each lane. Each sensor transmits a sequence of binary values indicating the presence of vehicles which is used for estimating vehicle flow, vehicle speed, vehicle classification, etc. For instance, inductive loop detectors are deployed at pre-specified points for traffic signal control as illustrated in [17]. In [17], we can find various deployment strategies of inductive loop detectors for various applications. For the accurate estimation of vehicle queue length, road sensors are to be deployed at sophisticatedly arranged points, which requires high installation and maintenance cost. Also, in order to supply power and allow communications, long cables are required to be installed along with sensors. Even with excluding the cabling cost, the high sensor installation and maintenance cost makes sensor deployment in all intersection areas infeasible. Wireless sensors can avoid cabling, but they have the drawback of short lifetime due to their power-constrained batteries. The lifetime of wireless sensors can be lengthened by implementing energy harvesting capability in wireless sensors which converts the vibrations induced by vehicles into energy.

Instead of using video cameras or sensors, the mechanisms utilizing GPS-mounted probe vehicles have been proposed for the estimation of the vehicle queue length [26,27,28,29,30,31,32,33]. Probe vehicles are special purpose vehicles designed for monitoring road traffic situations and collecting trajectory data. The performance of the probe vehicle-based approach is affected by the number of probe vehicles deployed on the road. The ratio of the number of probe vehicles to the total number of vehicles is called the penetration ratio of probe vehicles. Larger penetration ratio is better for achieving higher accuracy in terms of the vehicle queue length estimation. In the probe vehicle-based mechanisms, due to low penetration ratio of probe vehicles, one of the major issues is to enhance the accuracy based on the insufficient information from probe vehicles. Another issue is how to efficiently estimate vehicle queue length or traffic volume from the substantial data collected by probe vehicles. The main purpose of using probe vehicles is to collect traffic-related data throughout their journey and, then, to do the off-line analysis or estimation of traffic situations based on the collected data. Therefore, the probe vehicle-based approach is not for the real-time control of traffic signals.

The aim of our mechanisms differs from that of the probe vehicle-based mechanism in that our mechanisms use V2I communications for the real-time traffic signal control. That is, we consider the environment where the traffic signal controller detects vehicles through V2I communications and estimates the length of the vehicle queue and, then, controls the traffic signals. As the age of IoV is approaching [34,35], all the vehicles performing V2I communications (i.e., the penetration ratio of probe vehicles is 1) will be realized in the near future. In this case, V2I communication attempts from all the vehicles may cause collisions, so our objective is to limit the number of vehicles sending messages to traffic signal controllers without deteriorating the accuracy of the estimated vehicle queue length.

## 3. V2I Communications for Vehicle Queue Length Estimation

### 3.1. Communication Environment

**Figure 1:**
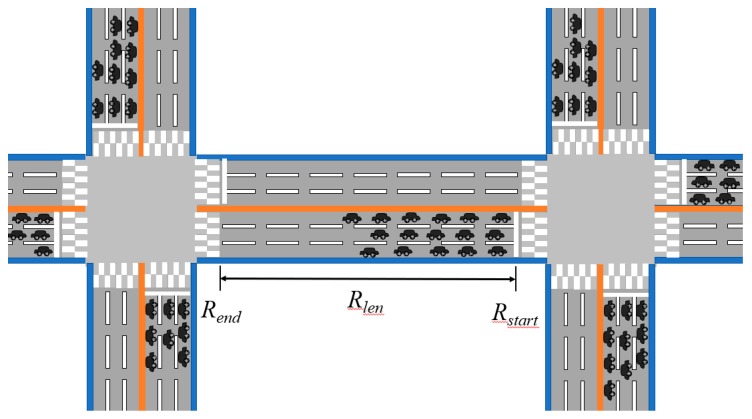
A road segment [22].

The road is composed of road segments each of which has vehicles heading to an intersection with a traffic controller. In this paper, we consider a road segment starting from $R_{start}$ to $R_{end}$ with the length of $R_{len}$ (see Figure 1). The vehicle queue is the queue of vehicles waiting for the green light and a vehicle decides that it is in the vehicle queue if its speed is lower than the specific speed at the red light. A vehicle in the vehicle queue is called an in-vehicle-queue (IVQ) vehicle and may send a Vehicle Information (VI) message to its upcoming traffic signal controller. The VI message has the information of the IVQ vehicle such as identifier, location, speed and moving direction. A traffic signal controller can estimate the vehicle queue length to control the traffic signal based on the received VI messages. We assume that the transmission range of a vehicle, $V_{range}$, is large enough to cover the upcoming traffic signal controller; that is, $V_{range}\geq R_{len}$. Vehicles move at a speed faster than the stopping speed when it is not in the vehicle queue, and know the information of traffic signal controllers and all the information related to the road segment, such as $R_{start}$, $R_{end}$, $R_{len}$, etc.

### 3.2. Distance-Based Transmission of Vehicle Information Messages

If we allow any vehicles in the vehicle queue to transmit VI messages (we call this the naïve mechanism), the number of VI message transmissions will be the same as the number of the vehicles in the vehicle queue. From the perspective of the accuracy in estimating the vehicle queue length, this mechanism is the best. However, this will lead to higher possibility of collisions. Therefore, we need to figure out the way of reducing the number of VI messages. The optimal way of achieving this is to allow only the last vehicle in the vehicle queue to transmit a VI message. However, a vehicle has no means of knowing that it is the last vehicle in the vehicle queue because it cannot know whether there are any vehicles following itself. If the V2V communication is adopted for that purpose, a vehicle can know whether there are any following vehicles or not. Even with the V2V communication, in the situation of contiguous vehicles running on the road, if a vehicle does not send a VI message because of any following vehicles, the transmission of a VI message may be delayed, resulting in non-reactive traffic signal control. Therefore, we proposed a mechanism, the distance-based mechanism, in which an IVQ vehicle sends a VI message according to its distance from the intersection in our prior work [17]. In the mechanism, we allow an IVQ vehicle farther from the upcoming intersection to send a VI message earlier and any IVQ vehicles closer to the intersection not to transmit any VI messages if they overhear a VI message behind themselves. For that, we introduced a timer that is used for an IVQ vehicle to defer a VI message transmission according to the distance from the intersection:

(1) $$T_{M}=T_{current}+\tau \times \frac{R_{len}-V_{dist}}{R_{len}}$$

**Figure 2:**
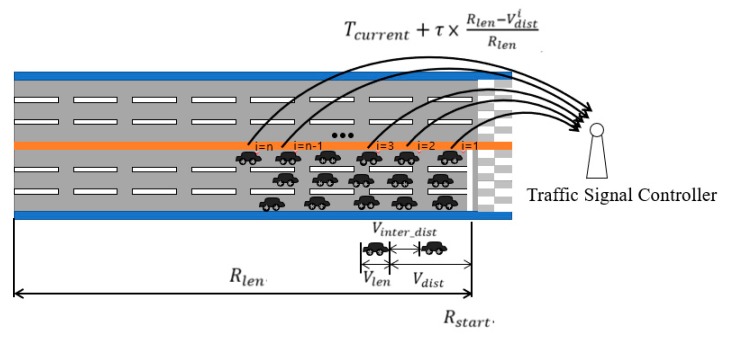
VI message transmissions of the distance-based mechanism [22].

An IVQ vehicle can transmit a VI message M at time $T_{M}$. $T_{current}$ is the current time and τ is the unit time and $V_{dist}$ is the distance of the vehicle from $R_{start}$. In Equation (1), the second term gives randomness to $T_{M}$ according to the distance from $R_{start}$ so that the collisions caused by simultaneous VI message transmissions can be avoided. Once an IVQ vehicle closer to the traffic signal controller listens a VI message from a farther IVQ vehicle, the closer IVQ vehicle gives up its transmission, resulting in less VI message transmissions. Figure 2 illustrates the operation of the distance-based mechanism from the perspective of VI message transmissions.

From the collected VI messages, the traffic signal controller estimates how many vehicles are in the vehicle queue. The estimated length, $Q_{len}$, of the vehicle queue is computed as follows:

(2) $$Q_{len}=\{\begin{array}{c} \underset{\forall M^{i}}{\max}\left[\left(⎡\frac{V_{dist}^{i}}{V_{len}+V_{inter\_dist}}⎤+1\right)\times k\right],\;if\;V_{dist}^{i}>0\\ 1\times k,\;otherwise \end{array}$$

Here, k is the number of lanes, $M^{i}$ is the i-th VI message and $V_{dist}^{i}$ is the distance of the vehicle sending $M^{i}$. $V_{len}$ is the length of a vehicle and $V_{inter\_dist}$ is the distance between two back-to-back vehicles. For simplicity, we assume that $V_{len}$ and $V_{inter\_dist}$ are constant.

However, at the red light, vehicles tend to line up one after another with slowing down their speeds with some time gap and may send VI messages to the traffic signal controller sequentially because of the larger time gap between the stopping times of two back-to-back vehicles compared with the random time delay gap between them determined according to the distance from the upcoming intersection. This may result in non-optimal VI message transmissions because of the VI message transmissions of the vehicles in the middle of the vehicle queue. Therefore, in the following subsection, we propose a new mechanism that can further reduce the VI message transmission overhead by limiting the areas in which vehicles are allowed to transmit VI messages.

### 3.3. Sector-Based Transmission of Vehicle Information Messages

**Figure 3:**
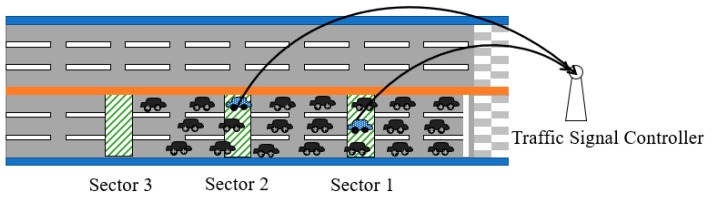
VI message transmissions of the sector-based mechanism.

The objective of the sector-based mechanism is to reduce the number of candidate IVQ vehicles to transmit VI messages. Compared with the distance-based mechanism in Section 3.2 where all the IVQ vehicles have the chances to transmit VI messages, the sector-based mechanism allows only the IVQ vehicles located in the sectors to transmit VI messages. Sectors are designated areas on a road segment. This is a reasonable approach because a vehicle tends to stop right behind a stopped vehicle and the fact that a vehicle V sends a VI message implies that the vehicles ahead of V have already stopped and belong to the vehicle queue. Figure 3 shows the operation of the sector-based VI message transmission mechanism. In the figure, each sector is represented as a square-shape area filled with slashes and the vehicles sending VI messages are filled with small dots.

In order to reduce the number of VI message transmissions from a sector, if an IVQ vehicle overhears the VI message transmission from another IVQ vehicle in the same sector, it gives up its transmission. The sector identifier is included in the VI message so that multiple VI messages with the same sector identifier cannot be transmitted.

The starting positions of sectors are determined by the sector length $S_{len}$ and the inter-sector distance $S_{inter\_dist}$. The starting location of the first sector, $S^{1}$, is $S_{start}$ meters away from $R_{start}$, the start position of the road segment. Then, the distance in meters from $R_{start}$ of the starting location of the ith sector $S^{i}$, $S_{start}^{i}$, is computed as follows:

(3) $$S_{start}^{i}=S_{start}+\left(i-1\right)\times \left(S_{len}+S_{inter_{dist}}\right),\;if\left(S_{start}^{i}+S_{len}\right)\leq R_{len}$$

**Figure 4:**
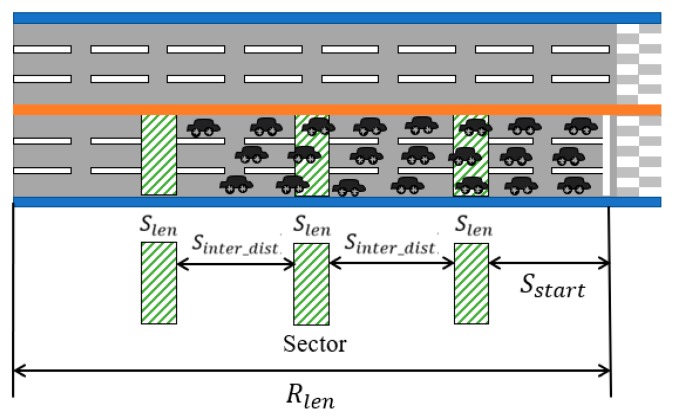
Sectors of the sector length $S_{len}$ and the inter-sector distance $S_{inter\_dist}$ with the first sector starting at the position $S_{start}$ meters from $R_{start}$.

Then, the number of sectors is the largest i that satisfies the condition $\left(S_{start}^{i}+S_{len}\right)\leq R_{len}$. Figure 4 shows how sectors are determined.

In the sector-based mechanism, the traffic signal controller collects VI messages and computes the length of the vehicle queue based on the collected VI messages, like in the distance-based mechanism. However, because there exists a gap (i.e., a non-sector area) between two consecutive sectors, the last vehicle in the vehicle queue may be located at the last sector, $S^{last}$, or in the non-sector area right before $S^{last+1}$. Therefore, we take the estimated vehicle queue length $Q_{len}$ to be the average of the minimum vehicle queue length $Q_{len}^{min}$ and the maximum vehicle queue length $Q_{len}^{max}$:

(4) $$Q_{len}=\{\begin{array}{c} \frac{Q_{len}^{min}+Q_{len}^{max}}{2}\times k,\;if\;V_{dist}^{last}>0\\ 1\times k,\;otherwise \end{array}$$

$$Q_{len}^{min}=⎡\frac{V_{dist}^{last}}{V_{len}+V_{inter\_dist}}⎤+1$$

$$Q_{len}^{max}=⎡\frac{S_{start}+i\times \left(S_{len}+S_{inter\_dist}\right)}{V_{len}+V_{inter\_dist}}⎤-1$$

**Figure 5:**
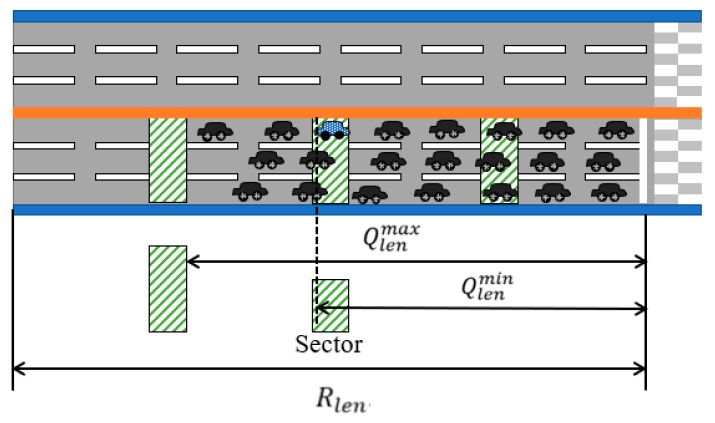
Vehicle queue length estimation of the sector-based mechanism.

Here, k is the number of lanes, $V^{last}$ is the IVQ vehicle in $S^{last}$ and $V_{dist}^{last}$ is the distance of $V^{last}$ from $R_{start}$. $Q_{len}^{min}$ is the vehicle queue length when $V^{last}$ is the last vehicle in the vehicle queue and $Q_{len}^{max}$ is the vehicle queue length when $V^{last}$ is located right before $S^{last+1}$. Figure 5 shows $Q_{len}^{min}$ and $Q_{len}^{max}$ of the case when the vehicle (with a dot pattern) on the first lane of the second sector sends a VI message to the traffic signal controller.

## 4. Performance Evaluation and Discussion

### 4.1. Simulation Environment and Performance Factors

Simulations were performed with the vehicular network simulation framework Veins [23] based on SUMO [24] and OMNet++ [25]. The IEEE 802.11p [21] is used as the MAC protocol for vehicular wireless communications and no background data traffic is generated except for VI message transmissions. The simulation network is a 4-way intersection with three lanes per road segment and the vehicle queue length is measured for a specific road segment. Three types of vehicles with different maximum speed and acceleration values are deployed for a realistic road traffic environment. The simulation parameters are listed in Table 1.

We evaluate and compare our distance-based and sector-based mechanisms with the Naïve mechanism for various simulation scenarios. The Naïve mechanism measures the vehicle queue length by making every IVQ vehicle to send a VI message to the traffic signal controller. The degree of saturation (%) is taken as one of the performance affecting factors, which is a ratio of demand (the number of vehicles moving on the road segment) to capacity (the maximum possible number of vehicles on the road segment) in percentage. We evaluate the performance of the proposed mechanisms for two degree of saturation scenarios, 30% and 50%. For the sector-based mechanism, the sector length $S_{len}$ and the inter-sector distance $S_{inter\_dist}$ are used as the performance affecting factors. For both $S_{len}$ and $S_{inter\_dist}$ values, 10 m, 20 m and 30 m are taken.

For the performance analysis, we measure three performance factors:

- The accuracy of the vehicle queue length estimation: The accuracy of the estimated vehicle queue length is measured in terms of the arithmetic mean (AM), the mean absolute deviation (MAD) and the mean absolute percentage error (MAPE). With a given data set, AM is obtained by dividing the sum of the given data set by the set size. MAD is the average of the absolute deviations from the mean (AM) of the given data set and MAPE is a measure of prediction accuracy in percentage. If $A_{i}$ and $F_{i}$ are the ith measured and estimated values, respectively, and n is the total number of measured values, the AM of $\left|A_{1}-F_{2}\right|,\ldots ,\;\left|A_{n}-F_{n}\right|$ is the average of the absolute deviations $\left|A_{1}-F_{2}\right|,\ldots ,\;\left|A_{n}-F_{n}\right|$:
  (5) $$AM=\frac{\sum_{i=1}^{n}\left|A_{i}-F_{i}\right|}{n}$$
  The MAD of $\left|A_{1}-F_{2}\right|,\ldots ,\;\left|A_{n}-F_{n}\right|$ is the average of the absolute deviations $\left|A_{1}-F_{2}\right|,\ldots ,\;\left|A_{n}-F_{n}\right|$ from the AM of $\left|A_{1}-F_{2}\right|,\ldots ,\;\left|A_{n}-F_{n}\right|$:
  (6) $$MAD=\frac{\sum_{i=1}^{n}\left|\left|A_{i}-F_{i}\right|-\frac{\sum_{j=1}^{n}\left|A_{j}-F_{j}\right|}{n}\right|}{n}$$
  and the MAPE of $A_{i}$’s and $F_{i}$’s is the average of $\left|\frac{A_{1}-F_{1}}{A_{1}}\right|$,…, $\left|\frac{A_{n}-F_{n}}{A_{n}}\right|$, expressed as a percentage:
  (7) $$MAPE\left(\%\right)=\frac{\sum_{i=1}^{n}\left|\frac{A_{i}-F_{i}}{A_{i}}\right|}{n}\times 100$$
- The number of VI message transmissions: This performance factor is used for measuring the message transmission overhead sent from the IVQ vehicles to the traffic signal controller. The number of VI message transmissions is measured by counting in the original transmissions and the retransmissions of VI messages during the simulation time.
- The VI message transmission delay: The transmission delay of a VI message is the time taken for a VI message to be delivered to the traffic signal controller successfully.

For the performance evaluation, we have executed five simulation runs for each mechanism and for each $S_{len}$ and $S_{inter\_dist}$ value pairs in the case of the sector-based mechanism. We use 10 m, 20 m and 30 m as the values of $S_{len}$ and $S_{inter\_dist}$.

### 4.2. Simulation Results

Table 2 lists the simulation results in terms of the vehicle queue length with the degree of saturation 30%. The number in the ‘Round’ column of the table indicates the execution order of the corresponding simulation run. The actual vehicle queue length is obtained from the Naïve mechanism and the estimated vehicle queue lengths from the distance-based and the sector-based mechanisms. The distance-based mechanism performs almost the same as the Naïve mechanism with less VI message transmissions (will be described later in this subsection). On the other hand, the sector-based mechanism shows 1.3~1.94 vehicle differences from the actual vehicle queue length, except for $S_{len}$ or $S_{inter\_dist}$ of 30 m. However, for the 3-lane road segment, 1~2 vehicle difference may not significantly affect the performance of the traffic signal controller. With considering only about 4 to 7.4 message transmissions of the sector-based mechanism (will be described later in this subsection), the sector-based mechanism is a good candidate for the vehicle queue length estimation because it significantly decreases the VI message transmission overhead.

In order to measure the accuracy of each of the proposed vehicle queue length estimation mechanisms, Table 3 shows the simulation results in terms of the AM and the MAD and the MAPE of the estimated vehicle queue length for the degree of saturation 30%. AM is the average of the absolute differences between the actual vehicle queue length and the estimated vehicle queue length. So, lower AM values mean better performance in estimating the vehicle queue length. From the MAD values in Table 3, we can anticipate the degree of stability of the mechanisms in estimating the vehicle queue length from the perspective of accuracy. As for MAD, smaller MAD values indicate better stability, so it can be asserted that the distance-based mechanism gives more stable estimated values than the sector-based mechanism. The sector-based mechanism performs well enough, except for $S_{len}$ = 30 m or $S_{inter\_dist}$ = 30 m. Setting $S_{len}$ or $S_{inter\_dist}$ to a larger value has a tendency to over- or under-estimate the vehicle queue length because of large variance in $Q_{len}^{min}$ and $Q_{len}^{max}$. MAPE is the average of the ratios of the difference between the actual and the estimated vehicle queue lengths to the actual vehicle queue length, represented in percentage. MAPE indicates the relative significance of the difference between the actual and the estimated vehicle queue lengths to the actual vehicle queue length. Even though the MAPE values of the sector-based mechanism are larger than those of the distance-based mechanism, this is acceptable because, for example, the MAPE value of 6.59% implies 0.659 vehicle difference per lane for the 3-lane road segment (0.659=(30×0.0659)÷3). Even for the worst case of $S_{len}$ = 30 m and $S_{inter\_dist}$ = 10 m, the MAPE value of 24.74% implies 2.474 vehicle difference per lane. We take the case of $S_{len}$ = 30 m and $S_{inter\_dist}$ = 10 m (i.e., the sector with 4 vehicles on a lane for the $V_{len}$ of 5 m and the $V_{inter\_dist}$ of 2.5 m) to show how badly the sector-based mechanism performs.

Table 4 shows the number of VI message transmissions and the average transmission delay of a VI message for the degree of saturation 30%. In the distance-based mechanism, the IVQ vehicles generate 21 VI messages in total and, in the naïve mechanism, 44. Thus, we can say that the distance-based mechanism is significantly better than the naïve mechanism in terms of the VI message transmission overhead. The transmission delay of a VI message is almost the same in all the mechanisms. The reason for this is that there is not sufficient traffic generated to hinder the transmissions of VI messages because there exist only the VI messages generated by the vehicles moving in one direction on a single road segment with no background traffic.

Table 5 shows the estimated vehicle queue length for the degree of saturation 50%. The distance-based mechanism works almost the same as the naïve mechanism in terms of the vehicle queue length estimation. Similar to the case of the degree of saturation 30%, the sector-based mechanism performs worse than the distance-based mechanism and the larger $S_{len}$ or $S_{inter\_dist}$ value gives worse performance. The sector-based mechanism shows 2.5~6.86 vehicle differences from the actual vehicle queue length. For the 3-lane road segment, 2~7 vehicle difference is acceptable with considering the actual queue length of around 50 vehicles.

Table 6 gives the accuracy values computed from the estimated vehicle queue lengths in Table 5. As for AM, MAD and MAPE, the distance-based mechanism performs better than the sector-based mechanism. The distance-based mechanism works almost the same as the naïve mechanism from the perspective of the accuracy in estimating the vehicle queue length. Even though the MAD and MAPE values of the sector-based mechanism are higher than those of the distance-based mechanism, for the 3-lane road segment, the MAD and the MAPE values are acceptable due to the same reasoning as that in Table 3. Similar to the case of the degree of saturation 30%, the sector-based mechanism performs worse than the distance-based mechanism and larger $S_{len}$ or $S_{inter\_dist}$ values give worse performance.

Table 7 shows the number of VI message transmissions and the average transmission delay of a VI message for the degree of saturation 50% The distance-based mechanism outperforms the naïve mechanism because the distance-based mechanism generates 37.4 VI message transmissions compared with 80 VI transmissions of the naïve mechanism, which is less than a half of the VI message transmission overhead of the naïve mechanism. Compared with 80 and 37.4 message transmissions of the naïve and the distance-based mechanisms, respectively, 8~12.8 message transmissions of the sector-based mechanism are desirable in the real-world environment with heavy data traffic. The message transmission overhead of the sector-based mechanism, with $S_{len}$ = 10 m and $S_{inter\_dist}$ = 10 m, is almost a third of that of the distance-based mechanism. With considering the message transmission overhead of the distance-based mechanism is less than a half of that of the naïve mechanism, the sector-based mechanism significantly outperforms the naïve mechanism. The transmission delay of a VI message is almost the same in all the mechanisms and the reason is the same as that for Table 4.

**Figure 6:**
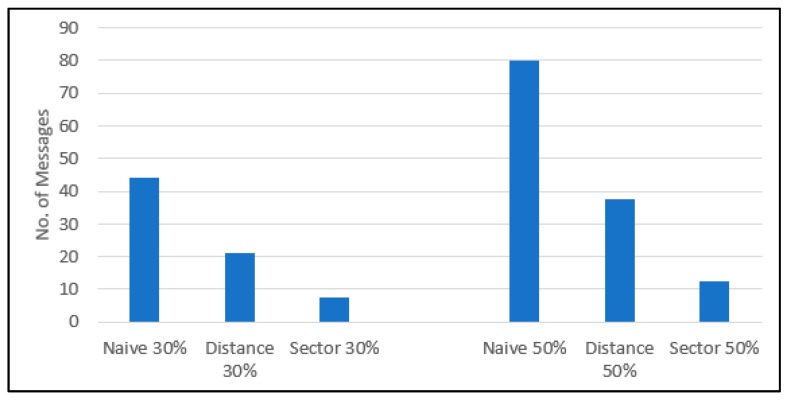
The VI message transmission overhead in terms of the number of VI messages transmitted with $S_{len}$ = 10 m, $S_{inter\_dist}$ = 10 m.

**Figure 7:**
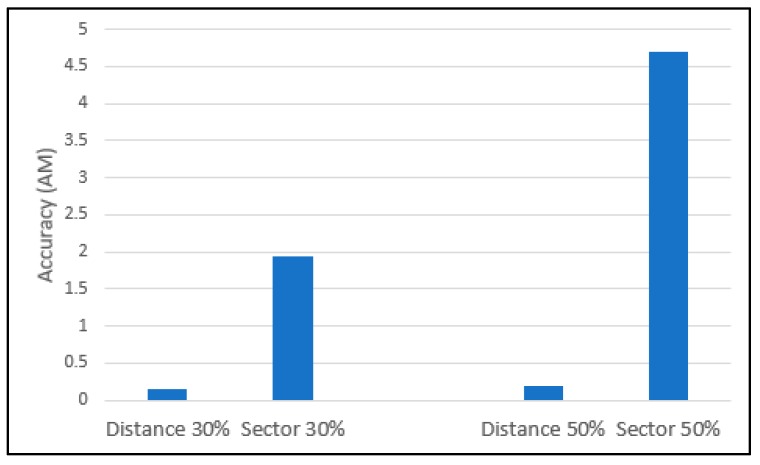
The accuracy of the estimated vehicle queue length in terms of AM with $S_{len}$ = 10 m, $S_{inter\_dist}$ = 10 m.

So far, we have analyzed the simulation results presented in tables for two cases of the degree of saturation. The common objective of the distance-based and the sector-based mechanisms is to estimate the vehicle queue length accurately with less message transmission overhead compared with the Naïve mechanism. Thus, we depict the accuracy in terms of AM and the number of VI message transmissions of the distance-based and the sector-based mechanisms in Figure 6 and Figure 7, respectively. In each graph of the figures, both the 30%- and the 50%-degree of saturation are plotted together in order to see how the degree of saturation has affected the performance.

In Figure 6, the V2I message transmission overhead is compared for three mechanisms in terms of the number of VI messages transmitted. The sector-based mechanism performs the best for both degree saturation cases and the performance improvement of our V2X communication-based mechanisms is not affected by the degree of saturation.

Figure 7 shows the estimation accuracy of our V2X communication-based mechanisms in terms of AM for two degree of saturation cases. The sector-based mechanism performs worse than the distance-based mechanism in all cases and the degree of saturation affects the performance of the sector-based mechanism more than that of the distance-based mechanism. According to the simulation results, as the degree of saturation increases, the number of vehicles in the vehicle queue increases and the average difference between the actual and the estimated vehicle queue lengths increases, too. From this, we can deduce that the number of vehicles in the vehicle queue affects the estimation accuracy of the sector-based mechanism. The reasoning behind this is that, in a large vehicle queue, the uncertainty of a vehicle being included in the vehicle queue increases because the action of a rear vehicle is influenced by the action of the vehicles ahead of the rear vehicle (more ahead vehicles in a longer vehicle queue). That is, a longer vehicle queue causes higher uncertainty of a rear vehicle (i.e., lower accuracy) especially in the sector-based mechanism because of the additional uncertainty caused by large variance in $Q_{len}^{min}$ and $Q_{len}^{max}$ of the sector-based mechanism.

### 4.3. Discussion

In the previous subsection, we have observed the microscopic performance of our V2X communication-based mechanisms in estimating the vehicle queue length for traffic signal control. In this subsection, we will discuss the pros and cons of our mechanisms in a broad sense, including comparisons with other relevant mechanisms. The aspects taken for the discussion are performance, required capabilities, operational cost, robustness, and extensibility:

- [Performance] As we have observed from the simulation results and analysis in Section 4.2, the distance-based mechanism achieves very high accuracy with very low (less than a half of the Naïve mechanism) message transmission overhead. Compared with the distance-based mechanism, the sector-based mechanism performs a slightly worse in terms of the estimation accuracy, but the discrepancy between the actual and the estimated values is acceptable as we pointed out in Section 4.2 regarding Table 3 and Table 6. The major merit of the sector-based mechanism is the vast reduction of the message transmission overhead which is almost a third of the distance-based mechanism and a sixth of the Naïve mechanism. We can say that our V2X communication-based mechanisms are well suited for road traffic situations. On the other hand, the sensor-based approach can hardly measure the right number of IVQ vehicles with sensors installed under the pavement at specific points of the road segment. This approach can only count the vehicles passing by sensors. As for the video-based approach, it has to cope with weather conditions, lighting and obtacles, like bent roads, trees and buildings, in order to correctly count vehicles.
- [Required Capabilities] Both the distance-based and the sector-based mechanisms require the GPS capability for the location information and a digital map for the intersection information and the V2X communication capability for VI message transmissions. The sector-based mechanism additionally requires the information on sectors. The capabilities required by both mechanisms are easily installable and do not require additional cost once vehicles are pre-equipped with GPS and V2X communication modules and digital maps. For situation awareness, smart vehicles are essentially equipped with GPS and V2X communication modules. Thus, both of our mechanisms are good for utilizing the intrinsic capabilities of smart vehicles. In the case of the sensor-based approach, sensors require communication capabilities like wires to transmit monitoring data to traffic signal controllers and the capabilities to lengthen the lifetime of sensors such as energy harvesting. For the video-based approach, the real-time processing capabilities are required to control traffic signals at the right time and the countermeasures for adversary environmental conditions are required to acquire video images with manageable qualities.
- [Operational Cost] Our V2X communication-based mechanisms require only the communication modules installed in vehicles which are intrinsic capabilities of smart vehicles for situation awareness. So, there is no additional installation and maintenance cost incurred solely by our mechanisms. Besides, the installation and the maintenance of the communication modules can be easily done only through vehicle inspections at vehicle manufacturing factories and vehicle maintenance centers. On the other hand, the video camera- and the sensor-based approaches require manual installation and maintenance of devices and/or communication links on the road, incurring a lot of efforts and costs.
- [Robustness] Our V2X communication-based mechanisms are resistant to harsh outside-world environments because they are not affected by weather, lighting and road pattern. The only obstacle is any objects hindering communications between vehicles and traffic signal controllers, but wireless communications are more resilient to obstacles than the line-of-sight nature of video cameras. With regard to robustness, the sensor-based approach is the weakest because sensors are susceptible to harsh road conditions like noise and vibrations. On the other hand, the V2X communication-based and the video-based approaches are not affected by road conditions. As for weather conditions, the video-based approach is the weakest because rain or snow will blur video images. Therefore, we can assert that our V2X communication-based approach is the best from the perspective of robustness.
- [Extensibility] Based on the vehicular information, like moving direction and speed, obtained from V2I communications, traffic signal controllers can cooperate for more enhanced control of traffic signals. On the other hand, the mechanisms using sensors are not capable of supporting cooperation among traffic signal controllers because there is no way of acquiring or deducing the direction of a vehicle. The video-based approach can guess the next road segment that a vehicle is heading towards via the lane on which the vehicle is. However, it has no way of knowing the route of a vehicle. On the contrary, our mechanisms may let traffic signal controllers know the routes of vehicles via VI messages so that they can collaborate on the optimization of traffic signal control in a wide area. By means of V2X communications, traffic signal controllers can collect various kinds of information from vehicles so that they can accomplish many valuable functionalities.

From the above discussion, we can conclude that the V2X communication-based approach is the best in estimating the vehicle queue length. The only limitation that we confront with is the penetration ratio of the vehicles equipped with V2X communication modules on the road. The optimistic point is that the immense efforts to realize the cooperative intelligent transport system (C-ITS) in Europe (known as the connected vehicle technology in the United States) [34,35] will help us in setting up the environment for our mechanisms. More specifically, the European Commission announced its plan for the coordinated deployment of C-ITS in Europe in order to start the full-scale deployment of C-ITS services and C-ITS enabled vehicles in 2019.

## 5. Conclusions

The Internet of Vehicles (IoV) allows vehicles to communicate with anything in the Internet, including vehicle themselves and traffic signal controllers on the road. In this paper, we focused on a cost-effective ITS application of controlling traffic signals in real-time in the IoV environment. Vehicular communication capabilities enable vehicles to communicate directly with traffic signal controllers (i.e., RSUs) located at intersections so that traffic signal controllers can estimate how many vehicles are waiting for the green light (i.e., vehicle queue length). Thus, with V2I communications, we can avoid using conventional computing-intensive video cameras or high operational-cost sensors for the real-time estimation of vehicle queue length. Furthermore, V2I communications are robust compared with video-taking and sensing in the harsh road environment with full of obstacles.

In the V2X communication-based approach, both the V2I communication overhead and the accuracy of the vehicle queue length estimation must be considered. For the reduction of V2I communication overhead, we proposed the distance-based mechanism in [17] and newly proposed the sector-based mechanisms in this paper. In the distance-based mechanism, vehicles farther from the traffic signal controller have higher possibility of sending messages to the traffic signal controller. In the sector-based mechanism, sectors are specified in a road segment and only the vehicle first stopped in each sector is allowed to perform V2I communications.

For the performance comparison of our mechanisms, we carried out simulations based on the Veins vehicle network simulation framework for various performance determining factors and analyzed the performance in terms of the message transmission overhead and the accuracy of the vehicle queue length estimation. The message transmission overhead indicates the utilization of constrained wireless link resource, so less message transmissions are preferred. From the simulation results, we showed that our mechanisms significantly reduce the number of message transmissions without losing the accuracy of the estimated vehicle queue length, compared with the Naïve mechanism. The sector-based mechanism decreases the message transmission overhead about a sixth of the Naïve mechanism and a third of the distance-based mechanism. This indicates that our V2X communication-based mechanisms are especially good for the road situation with many vehicles. Also, we verified that the proper selection of sector length and inter-sector distance is important for achieving acceptable performance of the sector-based mechanism. According to the simulation results, the sector length 10~20 m and the inter-sector distance 10~20 m are good enough for the case of the vehicle length 5 m and the inter-vehicle distance 2.5 m. Because sector length and inter-sector distance are easily tunable parameters, our sector-based mechanism can be applied to any road environments at no extra cost. Also, based on the discussion in Section 4.3, we come to the conclusion that our vehicular communication-based approach can be the ultimate enabler of intelligent traffic signal control in the age of IoV.

## Figures and Tables

**Table 1:** Simulation parameters.

Parameter	Setting
Network Size	570 m × 570 m
Road Segment Length ($R_{len}$)	250 m
Transmission Range of Vehicle	250 m
Vehicle Maximum Speed	60, 70, 80 km/h
Vehicle Acceleration	0.6, 0.8, 1.0 m/s^2^
Vehicle Deceleration	4.5 m/s^2^
Vehicle Length ($V_{len}$)	5 m
Inter-Vehicle Distance ($V_{inter\_dist}$)	2.5 m
Vehicle Stopping Speed	1 m/s
T	0.05 s
Transmission Range of RSU	250 m

**Table 2:** The estimated vehicle queue length (with degree of saturation = 30%).

Round	Actual Queue Length	Estimated Queue Length						
	Naïve	Distance-Based	Sector-Based ($S_{len}$ = 10 m)			Sector-Based ($S_{inter\_dist}$ = 10 m)		
			$S_{inter\_dist}$			$S_{len}$		
			10 m	20 m	30 m	10 m	20 m	30 m
1	30	30	26.8	29.5	23.9	26.8	29.5	35.5
2	30	30	27.5	29.5	23.9	27.5	30	35.5
3	28	28.6	27.5	29.5	23.9	27.5	29.5	35.5
4	26	26	27	29.5	23	27	29	36.9
5	30	29.8	27.5	29.5	23	27.5	28.1	35.5

**Table 3:** The accuracy of the estimated vehicle queue length (with degree of saturation = 30%).

Accuracy Measure	Distance-Based	Sector-Based ($S_{len}$ = 10 m)			Sector-Based ($S_{inter\_dist}$ = 10 m)		
		$S_{inter\_dist}$			$S_{len}$		
		10 m	20 m	30 m	10 m	20 m	30 m
AM	0.16	1.94	1.3	5.26	1.94	1.38	6.98
MAD	0.19	0.95	0.96	1.37	0.95	0.9	1.78
MAPE (%)	0.56	6.59	4.76	18.04	6.59	4.98	24.74

**Table 4:** The number of transmitted VI messages and the average transmission delay of a VI message for various inter-sector distances and sector lengths (with degree of saturation = 30%).

	Naïve	Distance-Based	Sector-Based ($S_{len}$ = 10 m)			Sector-Based ($S_{inter\_dist}$ = 10 m)		
			$S_{inter\_dist}$			$S_{len}$		
			10 m	20 m	30 m	10 m	20 m	30 m
No. of Messages	44	21	7.4	4	4	7.4	6.6	7.2
Delay (sec)	0.000239	0.00024	0.000238	0.000239	0.000243	0.000238	0.000239	0.00024

**Table 5:** The estimated vehicle queue length (with degree of saturation = 50%).

Round	Actual Queue Length	Estimated Queue Length						
	Naïve	Distance-Based	Sector-Based ($S_{len}$ = 10 m)			Sector-Based ($S_{inter\_dist}$ = 10 m)		
			$S_{inter\_dist}$			$S_{len}$		
			10 m	20 m	30 m	10 m	20 m	30 m
1	48	48	43	42	40	43	50.5	52.5
2	51	51	51.5	53.5	55.5	51.5	53.5	52.5
3	51	51	43	53.5	55.3	43	52	54
4	48	47	43	42	40	43	50.5	52.5
5	48	48	43	42	38.5	43	52	54

**Table 6:** The accuracy of the estimated vehicle queue length (with degree of saturation = 50%).

Accuracy Measure	Distance-Based	Sector-Based ($S_{len}$ = 10 m)			Sector-Based ($S_{inter\_dist}$ = 10 m)		
		$S_{inter\_dist}$			$S_{len}$		
		10 m	20 m	30 m	10 m	20 m	30 m
AM	0.2	4.7	4.6	6.86	4.7	2.5	3.9
MAD	0.32	1.68	1.68	1.97	1.68	0.6	1.32
MAPE (%)	0.42	9.58	9.46	14.08	9.58	5.12	8.01

**Table 7:** The number of transmitted VI messages and the average transmission delay of a VI message for various inter-sector distances and sector lengths (with degree of saturation = 50%).

	Naïve	Distance-Based	Sector-Based ($S_{len}$ = 10 m)			Sector-Based ($S_{inter\_dist}$ = 10 m)		
			$S_{inter\_dist}$			$S_{len}$		
			10 m	20 m	30 m	10 m	20 m	30 m
No. of Messages	80	37.4	12.6	9.4	8	12.6	12.8	12.8
Delay (sec)	0.00024	0.000241	0.000249	0.000239	0.000238	0.000249	0.000251	0.000251
